# Structural, Mechanistic, and Antigenic Characterization of the Human Astrovirus Capsid

**DOI:** 10.1128/JVI.02666-15

**Published:** 2016-02-11

**Authors:** Royce L. York, Payam A. Yousefi, Walter Bogdanoff, Sara Haile, Sarvind Tripathi, Rebecca M. DuBois

**Affiliations:** a Department of Biomolecular Engineering, University of California Santa Cruz, Santa Cruz, California, USA; b Department of Chemistry and Biochemistry, University of California Santa Cruz, Santa Cruz, California, USA

## Abstract

Human astroviruses (HAstVs) are nonenveloped, positive-sense, single-stranded RNA viruses that are a leading cause of viral gastroenteritis. HAstV particles display T=3 icosahedral symmetry formed by 180 copies of the capsid protein (CP), which undergoes proteolytic maturation to generate infectious HAstV particles. Little is known about the molecular features that govern HAstV particle assembly, maturation, infectivity, and immunogenicity. Here we report the crystal structures of the two main structural domains of the HAstV CP: the core domain at 2.60-Å resolution and the spike domain at 0.95-Å resolution. Fitting of these structures into the previously determined 25-Å-resolution electron cryomicroscopy density maps of HAstV allowed us to characterize the molecular features on the surfaces of immature and mature T=3 HAstV particles. The highly electropositive inner surface of HAstV supports a model in which interaction of the HAstV CP core with viral RNA is a driving force in T=3 HAstV particle formation. Additionally, mapping of conserved residues onto the HAstV CP core and spike domains in the context of the immature and mature HAstV particles revealed dramatic changes to the exposure of conserved residues during virus maturation. Indeed, we show that antibodies raised against mature HAstV have reactivity to both the HAstV CP core and spike domains, revealing for the first time that the CP core domain is antigenic. Together, these data provide new molecular insights into HAstV that have practical applications for the development of vaccines and antiviral therapies.

**IMPORTANCE** Astroviruses are a leading cause of viral diarrhea in young children, immunocompromised individuals, and the elderly. Despite the prevalence of astroviruses, little is known at the molecular level about how the astrovirus particle assembles and is converted into an infectious, mature virus. In this paper, we describe the high-resolution structures of the two main astrovirus capsid proteins. Fitting these structures into previously determined low-resolution maps of astrovirus allowed us to characterize the molecular surfaces of immature and mature astroviruses. Our studies provide the first evidence that astroviruses undergo viral RNA-dependent assembly. We also provide new insight into the molecular mechanisms that lead to astrovirus maturation and infectivity. Finally, we show that both capsid proteins contribute to the adaptive immune response against astrovirus. Together, these studies will help to guide the development of vaccines and antiviral drugs targeting astrovirus.

## INTRODUCTION

Human astroviruses (HAstVs) are a leading cause of viral gastroenteritis in children, the elderly, and immunocompromised patients (1–9), with approximately 3.9 million cases of HAstV gastroenteritis per year in the United States alone (10). There are eight canonical human serotypes, HAstV-1 through HAstV-8, and HAstV-1 is the most common serotype worldwide (1, 11, 12). Divergent strains of HAstV have been associated with encephalitis (13–15). The Astroviridae family also includes many nonhuman astroviruses (AstVs) that cause infections in mammals and birds, causing a range of symptoms, including gastroenteritis, fatal hepatitis, and neurological disease (16, 17).

**FIG 1:**
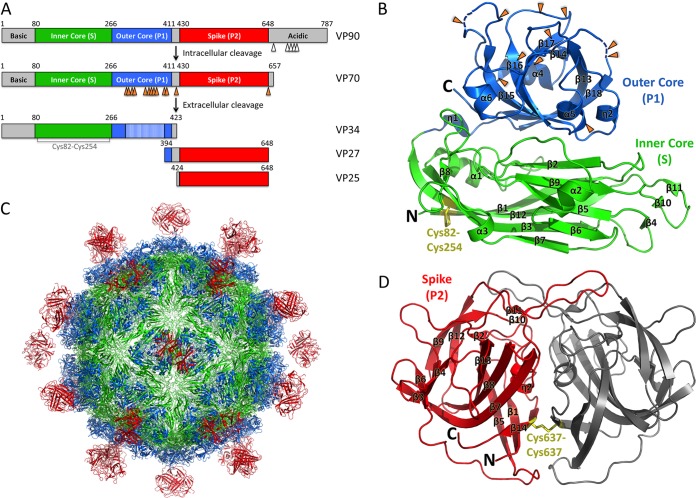
Schematics and crystal structures of HAstV-1 CP core and spike. (A) Schematics of the HAstV-1 CP domain structure and proteolytic processing events. Caspase and trypsin cleavage sites are indicated with white and orange arrows, respectively. (B and D) Crystal structures of the HAstV-1 CP core (B) and spike (D). Trypsin cleavage sites are indicated with orange arrows. Disulfide bonds are labeled and colored yellow. N and C termini are labeled. (C) Model of mature T=3 HAstV-1 virion. Figures were made with PyMOL.

AstVs are nonenveloped, positive-sense, single-stranded RNA viruses with three open reading frames (ORFs). ORF1a and ORF1b encode two nonstructural polyproteins (18, 19), and ORF2 encodes the capsid protein (CP) (20–22). The AstV CP is composed of several domains, including a highly basic N-terminal domain, a core domain, a spike domain, and a C-terminal acidic domain (Fig. 1A). Newly synthesized HAstV CP is 87 to 90 kDa (VP90) and assembles into immature HAstV particles inside infected cells (23, 24). HAstV CP undergoes a multistep maturation process via proteolytic cleavage events that are required for virus release and infectivity. First, intracellular caspases remove the C-terminal acidic domain of CP to generate VP70 (25, 26) (Fig. 1A). After immature HAstV release from cells, the CP is further processed by host extracellular proteases to produce mature HAstV. In cell culture, trypsin has been used to produce mature HAstV, whose infectivity is 10^5^-fold higher than that of immature HAstV not treated with trypsin (25, 27, 28). Mature HAstV is composed of three predominant proteins: VP34, VP27, and VP25 (25, 28–30) (Fig. 1A). The mechanism by which proteolysis increases HAstV infectivity is unknown. Mature HAstV was recently shown to gain entry into host cells via clathrin-mediated endocytosis (31).

Electron cryomicroscopy (cryo-EM) studies of immature and mature HAstV particles revealed an ∼35-nm T=3 icosahedral structural core projecting knob-like spikes (32). The immature HAstV particle shows remarkable resemblance to the T=3 hepatitis E virus (HEV) particle (32, 33). VP34 composes the structural core that forms a protein shell around the virus, and VP25 and VP27 form the spikes that protrude from the structural core. Comparison of immature and mature HAstV particles reveals a dramatic difference with respect to the surface spikes: immature HAstV particles contain 90 dimeric spikes, whereas mature HAstV particles contain only 30 spikes at the icosahedral 2-fold axes (32). Our lab and others previously determined the crystal structures of the AstV CP spike domains from HAstV-8 and turkey AstV-2 (TAstV-2) (34, 35); however, the structure of the CP core domain that comprises VP34 has remained elusive.

To further explore the structural features of the AstV CP, we determined the crystal structures of the HAstV-1 CP core and spike domains to 2.60-Å and 0.95-Å resolutions, respectively. Fitting of these structures into the cryo-EM density maps of immature and mature HAstV particles enabled characterization of the conserved molecular features on the surface of the virus. Our studies reveal new insights into the mechanisms of AstV particle assembly and maturation.

## MATERIALS AND METHODS

### HAstV-1 CP core production.

cDNA corresponding to HAstV-1 capsid protein residues 80 to 429 (accession number AAC34717.1) was cloned into pET52b in frame with a C-terminal thrombin cleavage site and a 10-histidine purification tag sequence. The plasmid was transformed into Escherichia coli strain BL21(DE3)pLysS, and HAstV-1 CP core expression was induced with 1 mM IPTG (isopropyl-β-d-thiogalactopyranoside) at 18°C for 16 h. A selenomethionine-substituted HAstV-1 CP core was expressed in E. coli strain B834(DE3)pLysS in M9 minimal medium supplemented with selenomethionine. E. coli cells were lysed by ultrasonication in 20 mM Tris, pH 7.5, 1 M NaCl, 5% (vol/vol) glycerol, 1 mM dithiothreitol (DTT), and 20 mM imidazole. The HAstV-1 CP core was purified from soluble lysates by HisTrap metal-affinity chromatography. The HAstV-1 CP core was further purified and characterized by size-exclusion chromatography on a Superdex 75 column in 10 mM Tris, pH 7.5, 1 M NaCl, 5% (vol/vol) glycerol, and 1 mM DTT. The pure HAstV-1 CP core was concentrated to ∼11 mg/ml.

### HAstV-1 CP core structure determination.

HAstV-1 CP core crystals were grown by the hanging-drop vapor diffusion method at 22°C in a well solution containing 1 M LiCl, 0.1 M sodium acetate, and 16.2% (vol/vol) polyethylene glycol 6000 (PEG 6000). Crystals were transferred to a cryosolution of well solution and 25% (vol/vol) ethylene glycol before being frozen in liquid nitrogen. Diffraction data were collected at cryogenic temperatures at Advanced Light Source beamline 5.0.1. Data were processed with HKL-2000 (36) (Table 1).

**TABLE 1:** Crystallographic statistics

Parameter	Value or description for the indicated crystal		
	HAstV-1 CP core	HAstV-1 CP core (SeMet)	HAstV-1 CP spike
Data collection parameters^*a*^			
Wavelength (Å)	0.97741	0.97939	1.03320
Space group	P2_1_2_1_2_1_	P2_1_2_1_2_1_	P4_1_2_1_2
*a*, *b*, *c* (Å)	66.24, 71.10, 158.91	66.56, 70.82, 159.06	103.23, 103.23, 41.67
α, β, γ (°)	90.0, 90.0, 90.0	90.0, 90.0, 90.0	90.0, 90.0, 90.0
Resolution (Å)	50.00–2.60 (2.69–2.60)	79.56–3.20 (3.42–3.20)	16.31–0.95 (1.0–0.95)
*R*_merge_	0.105 (0.558)	0.163 (0.434)	0.073 (0.460)
*I*/σ*I*	19.66 (3.41)	17.2 (7.0)	13.7 (3.1)
Completeness (%)	99.3 (98.4)	100 (100)	90.5 (67.4)
Redundancy	6.9 (6.9)	13.8 (14.3)	6.0 (4.5)
No. of selenium sites	NA	10	NA
Overall figure of merit	NA	0.31	NA
Refinement parameters			
Resolution (Å)	48.48–2.60		16.21–0.95
No. of reflections	23,493		141,071
*R*_work_/*R*_free_^*b*^	0.192/0.234		0.131/0.143
Ramachandran value (%)			
Favored	95.5		96.40
Allowed	4.5		2.88
Outliers	0.0		0.72
RMSD			
Bond lengths (Å)	0.017		0.008
Bond angles (°)	1.60		1.470

a Data were collected for a single crystal of each type. Values for the highest-resolution shell are shown in parentheses.

b *R*_free_ was calculated using 5% of the reflections.

The HAstV-1 CP core structure was solved by single-wavelength anomalous dispersion (SAD) (Table 1). Selenomethionine crystal data were collected at Advanced Light Source beamline 5.0.2 under cryogenic temperatures. Data were processed with HKL-2000 (36). Phenix (37) was used to determine the locations of 10 selenium sites and to autobuild an initial structural model. Although 14 sites were expected (7 per molecule in asymmetric units), 4 sites were not observed because they were located in flexible loops or position 1. Using the initial model and native data, the HAstV-1 CP core structure was refined and manually rebuilt by using Phenix (37) and Coot (38), respectively.

### HAstV-1 CP spike production.

cDNA corresponding to HAstV-1 capsid protein residues 429 to 645 (accession number AAC34717.1) was cloned into pET52b in frame with a C-terminal thrombin cleavage site and a 10-histidine purification tag sequence. The plasmid was transformed into Escherichia coli strain BL21(DE3), and HAstV-1 CP spike expression was induced with 1 mM IPTG at 18°C for 16 h. E. coli cells were lysed by ultrasonication in 20 mM Tris, pH 8.0, 300 M NaCl, 1 mM DTT, and 20 mM imidazole. The HAstV-1 CP spike was purified from soluble lysates by HisTrap metal-affinity chromatography. The HAstV-1 CP spike was further purified by size-exclusion chromatography on a Superdex 75 column in 10 mM Tris, pH 8.0, 150 mM NaCl, and 1 mM DTT. The pure HAstV-1 CP spike was concentrated to ∼18 mg/ml.

### HAstV-1 CP spike structure determination.

HAstV-1 CP spike crystals were grown by the hanging-drop vapor diffusion method at 22°C in a well solution containing 1 M ammonium sulfate, 6% PEG 400, and 0.1 M morpholineethanesulfonic acid (MES), pH 5.6. Crystals were transferred to a cryosolution of well solution and 35% (vol/vol) glycerol before being frozen in liquid nitrogen. Diffraction data were collected at cryogenic temperatures at Advanced Photon Source beamline 23-ID-B. Data were processed with HKL-2000 (36) (Table 1). The HAstV-1 CP spike structure was solved by molecular replacement, using the HAstV-8 CP spike structure (PDB ID 3QSQ) (34) and the program PHASER (39). The HAstV-1 CP spike structure was refined and manually rebuilt by using Phenix (37) and Coot (38), respectively.

### Modeling of HAstV-1 CP core and spike domains into the immature and mature HAstV cryo-EM maps.

The HAstV-1 CP core was structurally aligned to the three quasi-equivalent HEV CP molecules from the T=3 model determined previously by cryo-EM (33). This initial T=3 HAstV-1 CP core model was then fit into the 25-Å-resolution cryo-EM density map of HAstV-1 (32) by using the program Chimera (40). Assessment of the T=3 HAstV-1 CP core model revealed a number of intermolecular clashes between core domains. Thus, sequential fitting between the three quasi-equivalent HAstV-1 CP core molecules, in combination with symmetric fitting, was performed with Chimera to reduce the number of clashes. Sequential rounds of fitting of the three quasi-equivalent HAstV-1 CP core molecules into the cryo-EM map were then performed. This final T=3 HAstV-1 CP core model was used for construction of complete models of both immature and mature T=3 HAstV-1. To construct the immature T=3 HAstV-1 model, 30 HAstV-1 CP spike dimers at the icosahedral 2-fold symmetry axes and 60 HAstV-1 CP spike dimers at the icosahedral 5-fold vertices were fitted into the immature HAstV-8 cryo-EM density map (32). To construct the mature T=3 HAstV-1 model, 30 HAstV-1 CP spike dimers at the icosahedral 2-fold symmetry axes were fitted into the HAstV-1 cryo-EM map (32).

### Mapping of CP sequence conservation onto immature and mature T=3 HAstV-1 models.

The accession numbers for sequences used for sequence alignments of the AstV CP are as follows: HAstV-1 to -8, accession numbers O12792, Q82446, Q9WFZ0, Q3ZN05, Q4TWH7, Q67815, Q96818, and Q9IFX1; select mammalian astroviruses, accession numbers BAN62843, YP_006905854, ADH93577.1, YP_003090288.1, YP_006792628.1, YP_006905860.1, YP_006905857, ACX85472.1, ACX85474.1, ACX85476.1, AGO50636, Q80KJ6, BAA90309, AED89600.1, ADO67579.1, NP_059946.1, AII82243.1, CAR82567, ACR54274, AEM05826, ACF75865, AEV92822, YP_005271209.1, ADJ38391.1, ADX97522.1, and ACR54280.1; and select avian astroviruses, accession numbers YP_002728003, CBY02488, CBY02492, ADG45753, AEB15599, AEE88305, ABX46584, ABX46566, and AAV37187. Sequence alignments were performed by using Clustal Omega, and the conservation level was mapped onto the immature and mature T=3 HAstV-1 models by using Chimera (40).

### TAstV-2 CP core and spike production.

cDNAs corresponding to TAstV-2 CP residues 77 to 423 (core) and 424 to 631 (spike) (accession number NP_987088) were cloned, expressed, and purified using the same methods as those for the HAstV-1 CP core and spike, respectively. The only difference in methods was the use of E. coli strain Rosetta 2(DE3)pLysS for expression.

### ELISA.

Purified HAstV-1 or TAstV-2 CP core and spike (18.7 pmol/well) were incubated overnight at room temperature in their respective buffers in 96-well enzyme-linked immunosorbent assay (ELISA) microtiter plates. Plates were then washed three times with phosphate-buffered saline containing 0.05% Tween 20 (PBST). Wells were blocked by adding 150 μl Thermo Scientific StartingBlock blocking buffer, followed by three PBST washes. The blocking and washing steps were repeated a total of three times. Anti-HAstV-1 polyclonal rabbit serum (41) was initially diluted 1:20 with blocking buffer and then subsequently diluted 1:4 in series with blocking buffer. Wells were incubated with 150 μl of diluted polyclonal serum and incubated for 1 h at room temperature. Plates were washed three times with PBST and then incubated with a horseradish peroxidase (HRP)-conjugated secondary goat anti-rabbit IgG antibody diluted 1:5,000 in blocking buffer. Plates were washed three times with PBST and then developed by adding the peroxidase substrate *o*-phenylenediamine dihydrochloride (OPD) for 10 min at room temperature. The reactions were stopped with 3 M sulfuric acid, and the absorbance was measured at 490 nm.

### Protein structure accession numbers.

The HAstV-1 CP structural models and structure factors have been deposited into the online Protein Data Bank (PDB; www.pdb.org) as PDB entries 5EWN (CP core domain) and 5EWO (CP spike domain).

## RESULTS

### Structure of the HAstV-1 CP core domain.

Assuming that the HAstV-1 CP core would form an independently folded structural domain, we attempted to express it in Escherichia coli. An expression construct composed of HAstV-1 CP residues 80 to 429 produced a soluble protein and remained stable at high concentrations in buffers containing at least 1 M sodium chloride and 5% glycerol. The HAstV-1 CP core exhibited an apparent molecular mass of ∼20 kDa as examined by gel filtration chromatography, consistent with it being a compact monomeric protein in solution. Higher-molecular-mass peaks corresponding to possible capsomeres or virus-like particles were not observed; however, this may have been due to the high-salt and -glycerol buffer required to prevent protein precipitation.

**FIG 2:**
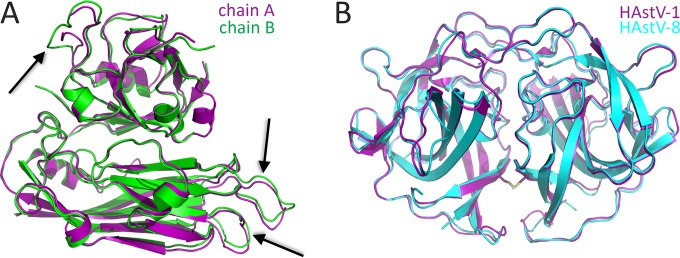
Structural alignments. (A) Alignment of the two molecules of HAstV-1 CP core in the asymmetric unit. Notable differences in the loops that form contacts at the 5-fold and 3-fold symmetry axes in the T=3 HAstV-1 model are indicated with arrows. (B) Alignment of HAstV-1 and HAstV-8 CP spike structures. The disulfide bond observed in the HAstV-1 CP spike is shown by stick models.

The purified HAstV-1 CP core was crystallized in the space group P2_1_2_1_2_1_, and a native diffraction data set was collected to 2.6-Å resolution (Table 1). Attempts to determine the structure by molecular replacement using the structure of the HEV CP S and P1 domains, which have only 18% sequence identity, or using homology models of the HAstV-1 CP core domain were unsuccessful, suggesting that there are structural differences. We therefore generated a selenomethionine-substituted sample and used single-wavelength anomalous dispersion methods to determine the 2.6-Å-resolution structure of the HAstV-1 CP core (Fig. 1B and Table 1). Two molecules in the asymmetric unit differ by a root mean square deviation (RMSD) of 1.0 Å, as assessed by the DaliLite server (42), with notable differences observed in the β4-β5, β10-β11, and α4-β15 loops that we predict are involved in intermolecular contacts in HAstV particle formation (see below) (Fig. 2A). Our final HAstV-1 CP core model contains amino acid residues 80 to 411, with a disordered loop at residues 390 to 396 being the only loop not visualized in at least one of the two molecules in the asymmetric unit (Fig. 1B). The DALI server (43) identified the HEV CP as the closest structural homolog, with an RMSD of 3.3 Å and a Z-score of 27.1 (with Z-scores of >2.0 being significant) (33, 44, 45).

### Structure of the HAstV-1 CP spike domain.

The HAstV-1 CP spike, composed of residues 429 to 645, was expressed in E. coli and purified. The HAstV-1 CP spike exhibited an apparent molecular mass of ∼50 kDa as examined by gel filtration chromatography, consistent with dimer formation. The purified HAstV-1 CP spike was crystallized in the space group P4_1_2_1_2, and a native diffraction data set was collected to 0.95-Å resolution (Table 1). The previously determined structure of the HAstV-8 CP spike was used as a molecular replacement model (34). Structural alignment of the HAstV-1 and HAstV-8 CP spike dimer structures by the DaliLite server (42) revealed nearly identical structures, with an RMSD of 0.8 Å (Fig. 2B). One noteworthy difference was an unexpected disulfide bond formed at the interface between the two protomers of the HAstV-1 CP spike dimer, despite the presence of reducing agents throughout protein purification (Fig. 1D). Sequence alignments of the HAstV-1 to -8 capsid proteins revealed that this disulfide bond is unique to serotype HAstV-1.

### Models of immature and mature T=3 HAstV-1 particles.

**FIG 3:**
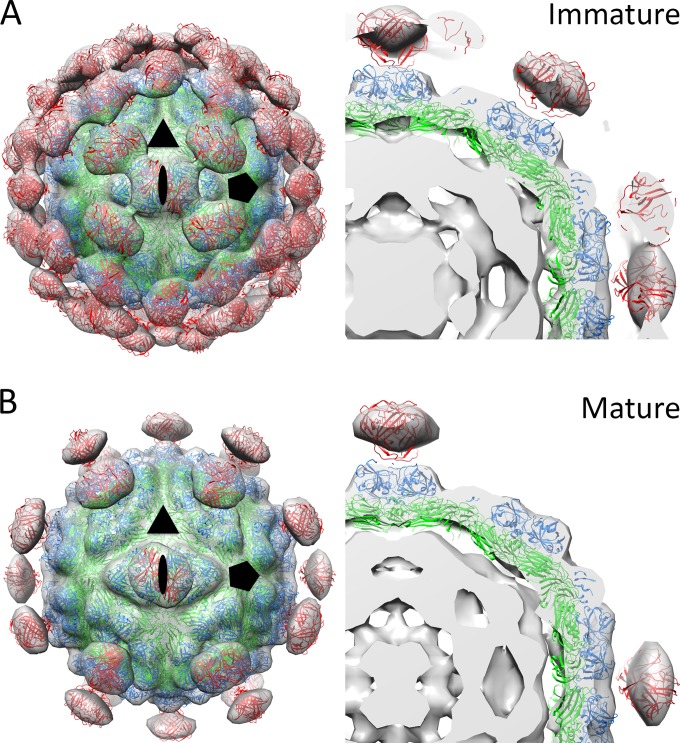
Immature and mature T=3 HAstV-1 models fit into HAstV cryo-EM maps. (A) Immature T=3 HAstV-1 model fit into HAstV cryo-EM map (left) and a zoomed-in, sliced view (right). (B) Mature T=3 HAstV-1 model fit into HAstV cryo-EM map (left) and a zoomed-in, sliced view (right). Models are shown as cartoon views and are colored as in Fig. 1, and cryo-EM maps are shown as gray transparent surfaces. An oval, a triangle, and a pentagon indicate the 2-fold, 3-fold, and 5-fold icosahedral axes, respectively. For immature HAstV, contour levels were set to encompass the expected volume of 180 copies of VP70 plus 1 copy of the viral RNA genome, i.e., ∼18.5 × 10^6^ Å^3^. For mature HAstV, contour levels were set to encompass the expected volume of 180 copies of VP34, 60 copies of VP25, and 1 copy of the viral RNA genome, i.e., ∼14.8 × 10^6^ Å^3^. Calculations of volume values are based upon a protein density of 1.35 g/cm^3^ and an RNA density of 2.0 g/cm^3^.

To investigate the molecular features on the surfaces of immature and mature HAstV particles, we first created an initial T=3 HAstV-1 CP core model by alignment with the three quasi-equivalent CP molecules from the T=3 HEV cryo-EM model (33) and fitting of this model into the 25-Å-resolution cryo-EM density map of HAstV-1 (32). We then performed iterative rounds of fitting of the three quasi-equivalent CP molecules into the cryo-EM density map by using the program Chimera (40). Using this T=3 HAstV-1 CP core model, we then added and fit HAstV-1 CP spike dimers into the protrusions of immature HAstV-8 and mature HAstV-1 cryo-EM density maps (32). Our final immature and mature T=3 HAstV-1 models show striking consistency with cryo-EM density maps (Fig. 3). The correlation coefficient measuring the agreement between a simulated map of our models at 25-Å resolution and the cryo-EM maps (also at 25-Å resolution) was 0.898 for immature HAstV and 0.902 for mature HAstV.

### Comparison of HAstV-1 and HEV CPs.

**FIG 4:**
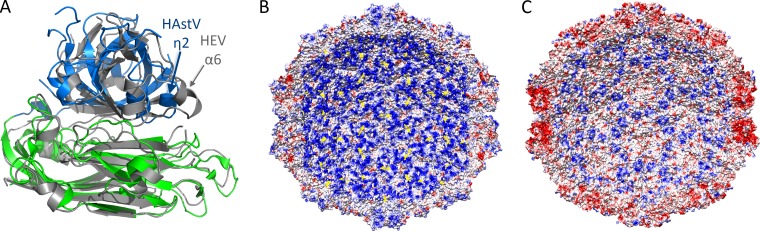
Structural and electrostatic comparisons between CP core domains from HAstV-1 and HEV. (A) Superposition of the HAstV-1 and HEV CP core domains. The HAstV-1 CP core is colored as in Fig. 1B, and the HEV CP core is colored gray. Significant differences between structures are indicated with arrows. (B and C) Electrostatic potential on the inner surface of the T=3 HAstV-1 CP core model (B) and the T=3 HEV CP core model (C). In the HAstV-1 model, the Cys82-Cys254 disulfide bond is colored yellow to show its proximity to electropositive residues. Note that in the T=3 HAstV-1 CP core model, the first 79 amino acids of the CP are not included. Similarly, in the T=3 HEV CP core model, the first 129 amino acids of the CP are not included. Thus, the electropositive surface comes only from amino acids on the inner core domain (S domain). Figures were made with Chimera (40).

The HAstV-1 CP core structure reveals two linear domains similar to the S and P1 domains of the HEV capsid (Fig. 1B and 4A). Structural alignment of the S domains (inner core) from HAstV-1 CP and HEV CP by use of the DaliLite server (42) revealed a Z-score of 20.0 and an RMSD of 2.4 Å (Fig. 4A). The S domains adopt a typical jelly roll β-barrel fold that is commonly found in CPs from small RNA viruses. Interestingly, another unexpected disulfide bond was observed in the HAstV-1 CP core, linking strands β1 and β12, despite the presence of reducing agents throughout purification, and this disulfide is conserved throughout serotypes HAstV-1 to -8 (Fig. 1B).

Structural alignment of the P1 domains (outer core) from HAstV-1 CP and HEV CP revealed a lower structural homology than that for the S domain, with a Z-score of 11.0 and an RMSD of 2.9 Å (Fig. 4A). While both P1 domains form a six-stranded β-barrel flanked by several α-helices, a 10-Å distance was observed between the HAstV-1 CP helix η2 and the equivalent helix α6 in HEV CP (Fig. 4A). As a result, HAstV has a more compact P1 domain than that in HEV.

To understand and compare the potential roles of viral RNA in interacting with CP and promoting virus assembly, we investigated the electrostatic potential on the inside surface of the T=3 HAstV-1 and HEV CP core models (Fig. 4). We observed a striking difference between the HAstV-1 and HEV surfaces, with HAstV-1 having a dramatically more electropositive surface. Notably, the disulfide bond observed in the HAstV-1 CP core (Fig. 1B) lies on the inner surface and may stabilize the nearby basic residues Arg84, Lys136, and Lys138 (Fig. 4B). These data suggest that the HAstV CP core may be directly involved in viral RNA binding.

### Locations of trypsin sites on HAstV-1 CP.

To better understand the composition of the CP in mature HAstV, we mapped the locations of trypsin cleavage sites onto the HAstV CP core P1 domain (Fig. 1B). Most of the 11 trypsin cleavage sites are exposed on surface-accessible loops, suggesting that many sites may become cleaved during maturation. Consistent with this idea, SDS-PAGE studies of purified mature HAstV-1 showed an absence of sizeable bands corresponding to the P1 domain (28, 32). However, the remarkable fit of the full HAstV-1 CP core structure into the cryo-EM map of mature HAstV-1 also suggests that trypsin cleavage does not entirely remove this region of the HAstV-1 CP.

### Conserved features on the surfaces of immature and mature AstV T=3 virions.

**FIG 5:**
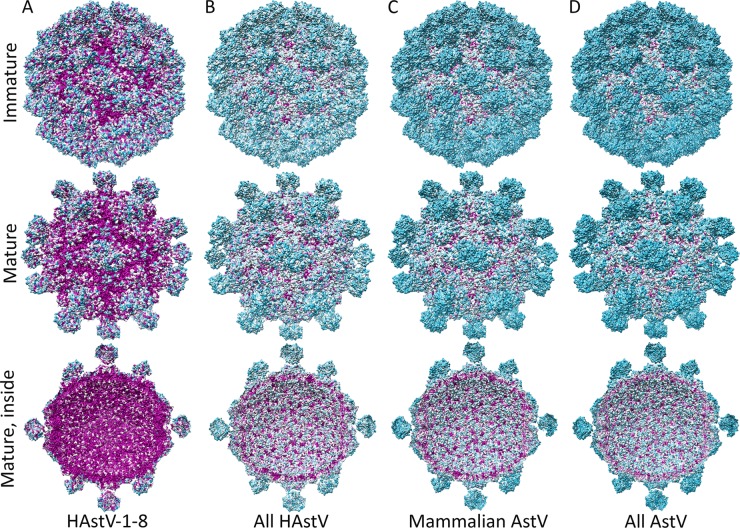
Conservation on the surfaces of immature and mature T=3 HAstV-1 models. Surface representations of T=3 immature and mature HAstV-1 models (top, bottom, and sliced in half) are shown. Residues conserved between canonical HAstV serotypes 1 to 8 (A), canonical and noncanonical HAstVs (B), mammalian AstVs (C), and mammalian and avian AstVs (D) are colored by level of conservation, from cyan (least conserved) to magenta (strictly conserved). Figures were made with Chimera (40).

To gain insight into the conserved features on the surfaces of AstVs that may play important functional roles in the AstV life cycle, we carried out multiple-sequence alignments and mapped the sequence conservation onto the immature and mature T=3 HAstV-1 models (Fig. 5). We first mapped out sequence conservation between canonical human serotypes HAstV-1 to -8 (Fig. 5A). There is a surprising lack of accessibility to conserved features on the surface of immature HAstV compared to mature HAstV. In addition, we observed almost complete sequence conservation on the inside surface of the virion, further supporting our electrostatic analyses showing that the inside surface of HAstV may play a key role in virus assembly.

We then mapped out sequence conservation between both canonical and noncanonical HAstV strains (Fig. 5B). The noncanonical HAstVs include the HAstV-MLB and HAstV-VA/HMO clades (14). The remarkable drop in surface conservation compared to that of canonical HAstVs highlights the evolutionary divergence between these HAstV strains. Indeed, these noncanonical HAstVs are more closely related to other mammalian AstVs than the canonical HAstVs, and this is apparent by the minimal changes in surface conservation observed by adding numerous other mammalian AstV CP sequences to the comparison (Fig. 5C). Finally, we mapped out sequence conservation between all AstV strains, including avian AstV strains (Fig. 5D). Although we observed high sequence divergence on the outside surface, there was a remarkable level of conservation remaining on the inside surface, again supporting the concept that the inside surface of AstVs may play a key role in binding viral RNA and promoting virus assembly and that this assembly mechanism may be conserved across all AstVs.

### Examination of polyclonal antibody binding and cross-reactivity.

**FIG 6:**
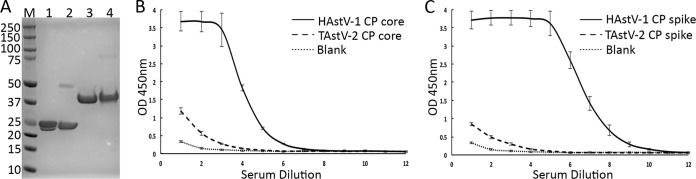
Reactivities of HAstV-1 and TAstV-2 CP core and spike domains to anti-HAstV-1 polyclonal rabbit serum. (A) Reducing SDS-PAGE analysis of purified proteins used for ELISA. Lanes: M, Bio-Rad Precision Plus molecular weight markers; 1, HAstV-1 CP spike; 2, TAstV-2 CP spike; 3, HAstV-1 CP core; 4, TAstV-2 CP core. Each lane was loaded with 10 μg protein. (B) Comparison of reactivities of purified HAstV-1 CP core (solid line) and TAstV-2 CP core (dashed line) to anti-HAstV-1 polyclonal rabbit serum by ELISA. (C) Comparison of reactivities of purified HAstV-1 CP spike (solid line) and TAstV-2 CP spike (dashed line) to anti-HAstV-1 polyclonal rabbit serum by ELISA. Serum dilution 1 was 1/20 in blocking buffer, and each subsequent dilution was obtained by a 1:4 series dilution with blocking buffer.

Almost nothing is known about the locations of epitopes on the surfaces of AstVs. Only two reports describe the isolation of anti-HAstV monoclonal antibodies, and all were found to immunoprecipitate VP25 or VP27, which we now know compose the HAstV spike (29, 41). To investigate the antigenicity of both the HAstV-1 CP core and spike, we performed ELISAs and assessed binding to anti-HAstV-1 polyclonal antibodies, which exhibit high neutralizing activity against HAstV-1 (41) (Fig. 6). Consistent with previous studies with monoclonal antibodies, we found that the HAstV CP spike is antigenic. Additionally, we showed that the HAstV CP core is also antigenic, though to a lesser extent. These data indicate that the mature HAstV-1 virion contains epitopes in both the CP core and spike domains that elicit an antibody response.

To investigate the cross-reactivity of anti-HAstV-1 polyclonal antibodies to divergent AstVs, we performed ELISAs with recombinant TAstV-2 CP core and spike (Fig. 6). We observed very little binding to anti-HAstV-1 polyclonal antibodies, suggesting a low level of sequence conservation between epitopes on HAstV-1 and those on TAstV-2.

## DISCUSSION

We report here the crystal structures of the HAstV-1 CP core and spike domains at 2.60-Å and 0.95-Å resolutions, respectively. By using these structures to model immature and mature T=3 HAstV-1 particles, we provide new molecular insights into HAstV assembly, maturation, immunogenicity, and evolution.

We provide new evidence that HAstVs and HEVs are phylogenetically related and may share commonalities in RNA-driven virus particle assembly. We found that the HAstV-1 CP core is structurally related to the HEV CP core, despite only 18% sequence identity between them. While interaction of the electropositive N terminus of HEV CP with viral RNA is a known driving force for T=3 HEV particle formation (44), little is known about the role of RNA in HAstV particle assembly. Here we observed a striking electropositive charge on the inside surface of T=3 HAstV particles, adding to the already highly positively charged N-terminal 79 residues that precede the HAstV CP core. The inside surface is highly conserved in HAstV-1 to -8 and even retains partial conservation throughout all AstVs. Previous studies of recombinant HAstV-1 CP, either full length or lacking the first 70 amino acids, revealed virus-like particle formation, suggesting that viral genomic RNA is not necessary for particle formation (23, 24). However, in these studies, the nucleic acid content of these virus-like particles was not examined, and we hypothesize that these particles were stabilized by cellular RNA bound to the electropositive charge on the inside surface. Consistent with this, we observed that recombinant HAstV-1 CP has a high absorbance at 260 nm (consistent with the sample containing nucleic acid) and precipitates upon addition of RNase but not DNase (data not shown). Thus, the structural similarities with HEV, the highly electropositive charge, and the high level of conservation inside AstV particles support a model in which binding of viral RNA to the AstV CP would drive T=3 AstV particle formation.

Our studies also provide new insights into HAstV maturation and the composition of mature T=3 HAstV particles. Like many viruses, HAstVs undergo proteolytic processing to produce mature, infectious particles. Although the *in vivo* protease(s) responsible for HAstV proteolysis has yet to be identified, it has been found that trypsin will increase HAstV infectivity 10^5^-fold in cell culture (25, 27, 28). N-terminal sequencing studies previously identified the trypsin cleavage sites at residues 494 and 324 that produce the VP25 and VP27 proteins of the HAstV-1 CP spike, respectively (25, 29); however, the boundaries of VP34 and the composition of the CP core domain in mature HAstV-1 remain elusive. Our data support a model in which the N terminus of the HAstV CP core is bound to RNA and protected from trypsin cleavage, making HAstV CP residue 1 the beginning of VP34. Thus, the C terminus of VP34 is likely made by a trypsin cleavage site occurring after residue 300, resulting in the observed ∼34-kDa band on SDS-PAGE (25, 28, 29). Here we mapped all 11 trypsin cleavage sites occurring between residues 299 and 394 of the HAstV-1 CP core. Most of the sites lie on surface-exposed loops, suggesting their susceptibility to trypsin digestion; however, we do note that the trypsin cleavage sites at residues 299 and 304 lie on the side of the CP core on helix α4 and may be less susceptible. Thus, we hypothesize that the earliest trypsin cleavage occurs at R313, which is conserved in HAstV-1 to -8 and would result in a 33.7-kDa protein band. We also hypothesize that other trypsin cleavages occur; however, the remarkable fit of the full HAstV-1 CP core structure into the cryo-EM map of mature HAstV-1 suggests that trypsin cleavage does not remove this region of the HAstV-1 CP entirely. Instead, these data together suggest that the trypsin-matured HAstV1 CP P1 domain exists as proteolytic fragments that remain bound together through hydrophobic core interactions and the hydrogen-bonding network of the β-barrel.

**FIG 7:**
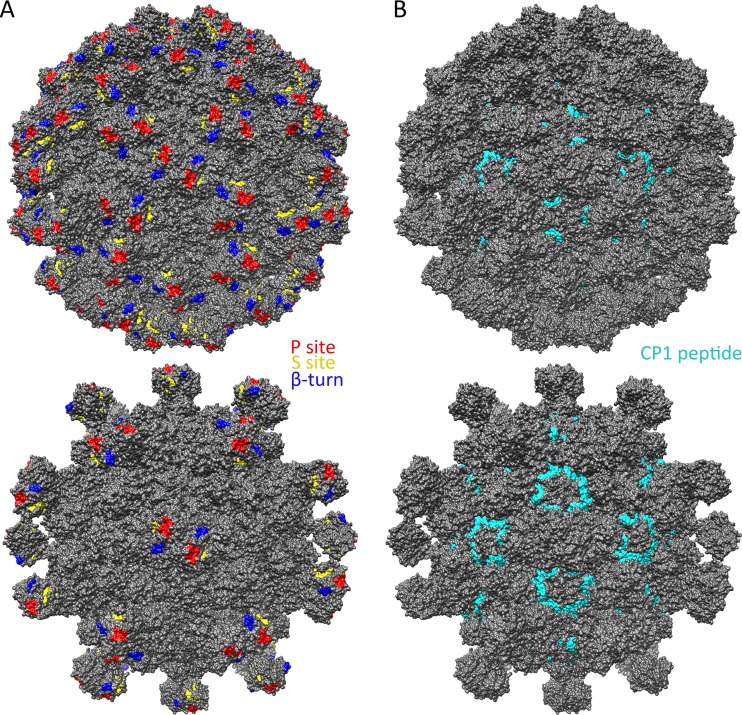
Known and predicted functional sites on the surfaces of immature and mature T=3 HAstV-1 models. (A) Predicted receptor-binding sites, i.e., the P site (red), the S site (yellow), and the β-turn (blue), mapped onto immature (top) and mature (bottom) T=3 HAstV-1 models. (B) The CP1 peptide (HAstV-1 CP residues 80 to 138) (cyan) that binds complement C1q, mapped onto immature (top) and mature (bottom) T=3 HAstV-1 models. Figures were made with Chimera (40).

Although the mechanism by which HAstV maturation by proteolysis increases infectivity is still unknown, our data provide insight into several possibilities, as follows. (i) Maturation may expose a receptor-binding site at an optimal time in the virus life cycle. Our data show that the surfaces of immature viruses are highly variable between HAstV-1 to -8, and maturation by trypsin, which removes 60 spikes, exposes a number of conserved sites on both the spike and core domains. A previous report identified three conserved potential receptor-binding sites on the spike domain: the P site, the S site, and the β-turn (34). We mapped these sites in the context of our immature and mature HAstV models and found that the P site lies on the most outward side of the virus and is exposed in both immature and mature HAstV (Fig. 7A). Thus, if the P site comprises a receptor-binding site, then immature HAstV would likely have the ability to attach to cells. In contrast, both the S site and the β-turn lie on the side of the spike and are sterically hindered by other spikes on immature HAstV. Thus, if the S site or β-turn comprises a receptor-binding site, immature HAstV would not likely have the ability to attach to cells, and receptor binding would not occur until after virus maturation. Although the true site of receptor binding is unknown, having it unexposed until an optimal time could be beneficial for both promoting virus release from cells and evading broadly neutralizing antibodies. (ii) Maturation may promote virus uncoating. Although the mechanism by which AstV is uncoated is unknown, protease maturation may change the ability of AstV to be triggered for uncoating in the endosome and for release of viral RNA. One possible mechanism is that the mature virus particle becomes less stable, making it primed for uncoating. We observed in cryo-EM maps the presence of electron density between spike dimers of immature HAstV, but not mature HAstV, suggesting that spike-spike interactions may stabilize the immature form (Fig. 3). (iii) Maturation may dampen host immunity. One possible mechanism is that the protease-released AstV spikes serve as decoys to dampen the effect of immune system antibodies. Another possible mechanism is that maturation allows HAstV to inhibit the complement pathway, and mapping of the previously determined C1q binding residues (HAstV-1 CP residues 80 to 138; also known as the CP1 peptide) revealed that they are exposed only on mature HAstV (46, 47) (Fig. 7B). Although these may play a role in AstV pathogenesis, it is unlikely the sole purpose of protease maturation given its dramatic effect on AstV infectivity.

Finally, our studies reveal for the first time that the HAstV CP core domain plays a role in the adaptive immune response to HAstV. We showed that polyclonal antibodies raised against mature HAstV-1 recognize both recombinant HAstV CP core and spike. Furthermore, we showed that these antibodies have almost no binding to a recombinant CP core and spike from an avian astrovirus (TAstV-2). These findings are consistent with the low level of conservation on the AstV particle surface observed by mapping out sequence conservation between mammalian and avian AstVs. Together, these antigenic studies have important implications for AstV vaccine development.
